# Clinical Characteristics and Outcomes of Culture-positive Versus Culture-negative Bone and Joint Infections in Pediatric Patients: A 10-Year Multicenter Study From Hungary

**DOI:** 10.1097/INF.0000000000005137

**Published:** 2026-01-05

**Authors:** Szofia Hajósi-Kalcakosz, Erzsébet Varga, Dorottya Őri, Márta Kovács, Kinga Nagy, Bence Horváth, Zoltán Nyul, Vera Piegl-Gulácsy, Gábor Fülep, Zsuzsanna Ökrös, Andrea Varannainé Soós, Csaba Ráskai, Beáta Visy, Ferenc Fekete, Bálint Gergely Szabó

**Affiliations:** * Department of Infectious Diseases, Heim Pál National Pediatric Institute, Budapest, Hungary; † Faculty of Medicine, Tropical Medicine Specialization, Semmelweis University, Budapest, Hungary; ‡ Heim Pál National Pediatric Institute, Budapest, Hungary; § Institute of Behavioral Sciences, Semmelweis University, Budapest, Hungary; ¶ Department of Pediatrics, Petz Aladár University Teaching Hospital of Győr-Moson-Sopron County, Győr, Hungary; ‖ Department of Pediatrics, University of Pécs, Pécs, Hungary; ** Department of Pediatrics, Szent György Hospital of Fejér County, Székesfehérvár, Hungary; †† Szent-Györgyi Albert Pediatric Clinic and Centre for Child Health, University of Szeged, Szeged, Hungary; ‡‡ Department of Pediatrics, Jósa András Hospital of Szabolcs-Szatmár-Bereg County, Nyíregyháza, Hungary; §§ Department of Orthopedic Surgery, Heim Pál National Pediatric Institute, Budapest, Hungary; ¶¶ Department of Internal Medicine and Haematology, Departmental Group of Infectious Disease, Semmelweis University, Budapest, Hungary; ‖‖ Doctoral College, Semmelweis University, Budapest, Hungary; *** South Pest Central Hospital, National Institute of Haematology and Infectious Diseases, Budapest, Hungary.

**Keywords:** bone and joint infection, arthritis, osteomyelitis, children, pediatric

## Abstract

**Background:**

Despite advancements, a proportion of pediatric bone and joint infections remain culture negative, complicating diagnosis and therapy. This study aimed to compare characteristics and outcomes of culture-negative versus culture-positive infections in a pediatric cohort over a 10-year period.

**Methods:**

A retrospective, multicenter observational study was conducted across 6 tertiary care centers in Hungary between 2015 and 2024, including hospitalized children with primary hematogenous osteomyelitis or septic arthritis within 2 months of symptom onset. The primary outcome was the requirement for surgical intervention within 30 days of diagnosis; secondary outcomes included the incidence of short-term (at 30 days) and long-term complications (at follow-up end). Postdischarge follow-up continued until the last documented hospitalization or outpatient visit.

**Results:**

Ninety-seven patients were included. Osteomyelitis, arthritis and combined infection were diagnosed in 49.5%, 28.9% and 21.6% of cases, respectively. A pathogen was identified in 72.5% of patients, most frequently via intraoperative cultures (74.2%) and blood cultures (53.6%). *Staphylococcus aureus* was the predominant pathogen (71.2%), followed by *Streptococcus pyogenes* (11.9%) and *Salmonella* spp. (6.8%). Within 30 days of diagnosis, the culture-positive group exhibited a significantly higher rate of surgical intervention, compared to the culture-negative group (79.7% vs. 34.2%, *P* = 0.001). Short-term complications were also more frequent in culture-positive cases (64.4% vs. 42.1%, *P* = 0.031). No difference was observed in long-term complication rates between groups.

**Conclusions:**

In pediatric patients, culture-positive bone and joint infections are associated with a more severe short-term clinical course and an increased likelihood of surgical intervention, particularly when *S. aureus* is the causative agent.

Pediatric bone and joint infections (BJIs) are severe conditions that require timely diagnosis and appropriate management to prevent potential orthopedic complications. Despite advancements in diagnostic techniques, including imaging and microbiologic methods, a considerable proportion of cases remain culture negative (CN), complicating the administration of targeted therapy. Understanding the distinctions between culture-positive (CP) and CN infections is essential for optimizing management strategies and improving patient outcomes.^1^

As pediatric BJIs are typically monomicrobial, identification of the causative pathogen is generally achieved through standard culture techniques, involving the isolation and identification of bacteria from blood and the infected site. Unfortunately, blood cultures are only capable of identifying a pathogen in less than half of all cases. Despite evidence that concurrent sampling from infected tissues increases the likelihood of identifying the causative pathogen, culture results remain negative in up to 70% of cases.^2^ The age and sex distributions of CP and CN cases are generally comparable, making it challenging to predict, at the time of diagnosis, which children are likely to develop CN BJI.^3^ For example, a study by Jia et al.^4^ identified several independent risk factors associated with negative culture results, including a symptom duration of more than 6.5 days, a maximum body temperature below 38.35°C at onset and a serum C-reactive protein (CRP) level below 78.40 mg/L.

The identification of the causative microorganism is crucial for guiding targeted antibiotic therapy. Among CP cases, methicillin-sensitive *Staphylococcus aureus* (MSSA) remains the predominant pathogen in Europe, accounting for approximately 30%–63% of microbiologically confirmed infections.^5^ Other notable pathogens include *Streptococcus pyogenes*, *Streptococcus pneumoniae* and *Kingella kingae*, particularly in children 6 months to 4 years old.^6–8^ Moreover, research has indicated that pediatric patients with CN BJIs frequently demonstrate a paucity of symptoms at diagnosis, including fever, high initial serum CRP, a high erythrocyte sedimentation rate and blood leukocytosis.^2^ Consequently, these patients may only require a reduced number of surgical interventions compared with those with CP BJIs. In addition, CN cases have been associated with a shorter treatment course and a shorter length of hospital stay.^3^ In accordance with these favorable short-term differences, a recent study by Chen et al.^2^ demonstrated that the incidence of long-term sequelae was significantly lower among patients with CN infections than among those with CP infections.

Therefore, we conducted a study to compare the clinical characteristics and outcomes of children with CN versus CP BJIs. We hypothesized that CN patients would present with less frequent fever, lower inflammatory marker levels at admission, a reduced need for surgical intervention and a lower incidence of long-term complications.

## MATERIALS AND METHODS

### Study Design and Settings

A retrospective, multicenter observational cohort study was conducted between January 1, 2015, and June 1, 2024, across 6 tertiary-referral pediatric centers of Hungary: Budapest (40 beds, 1.67 million estimated children in the coverage area), Győr (15 beds, 84,000 children), Nyíregyháza (15 beds, 103,000 children), Pécs (15 beds, 139,000 children), Szeged (18 beds, 200,000 children) and Székesfehérvár (17 beds, 127,000 children). The study population included pediatric patients (≤18 years of age at diagnosis) who were hospitalized with acute hematogenous osteomyelitis (AHO) or septic arthritis (SA) within 2 months of symptom onset, without a history of penetrating wound infection or orthopedic surgery. The exclusion criteria were as follows: (1) symptoms for ≥2 months, (2) penetrating wound infection, or previous surgical intervention at the affected site. The study was conducted in accordance with national ethical guidelines and the Declaration of Helsinki. Ethical approval was granted by the Hungarian National Medical Research Council (BM/22249-3/2024). Written informed consent was waived for this type of study.

### Data Collection and Study Definitions

Data from all included patients were anonymously recorded in a presolidated case report form. The following data were collected: (1) age, sex, comorbidities and symptoms at diagnosis; (2) laboratory, microbiologic and radiologic results at diagnosis and during hospitalization; (3) complications and surgical interventions during hospitalization; and (4) clinical outcomes and follow-up data. The diagnostic appropriateness of AHO and SA was validated by the principal investigators (S.H.K. and B.G.S.) during patient inclusion, with each investigator blinded to the other’s decision. The selection of cases for inclusion in the study was determined by the consensus of both principal investigators.

Definitions of AHO and SA were based on the European Society of Pediatric Infectious Diseases and the Infectious Diseases Society of America guidelines.^9–11^ Briefly, AHO was defined as^1^ fever, soft tissue swelling with warmth or bone pain with limited joint mobility (2 minutes), and^2^ radiologic features of pathologic bone resorption, subperiosteal/intraosseal abscess or necrosis, or bone sequestration (1 minute, on ≥1 imaging, such as radiograph, ultrasonography or magnetic resonance imaging [MRI]), with or without^3^ isolation of a causative pathogen from a blood or bone sample. SA was defined as^1^ fever, joint swelling and warmth, pain and limited mobility (2 minutes),^2^ joint effusion on ultrasonography, with or without^3^ isolation of a causative pathogen from blood or joint fluid. Complicated AHO was defined if an intraosseal abscess or necrosis was shown during radiologic or intraoperative visualization.

Both AHO and SA were categorized as acute if the symptom duration was 14 days or less at the time of hospitalization and as subacute if the duration ranged between 14 days and 2 months. Short-term complications of AHO and SA were defined as the presence of any of the following: myositis/pyomyositis, pneumonia, hepatic abscess, prolonged bacteremia (defined as at least two positive blood cultures taken at ≥72 hours apart), venous thrombosis, septic thrombophlebitis, infective endocarditis, involvement of ≥2 bones, epiphyseal plate involvement or pathologic fracture. Long-term complications were defined as pathologic fracture, limb length discrepancy, axial misalignment, restricted joint mobility and chronic osteomyelitis. We defined chronic osteomyelitis as typical clinical symptoms (see earlier) that had lasted for more than 2 months since diagnosis.

### Clinical Outcomes and Follow-Up

The primary clinical outcome was the requirement for surgical intervention within 30 days postdiagnosis. The secondary outcomes included the occurrence of short-term complications within the same 30-day period and the development of any long-term complications by the end of follow-up. Postdischarge follow-up was performed by a pediatric orthopedic specialist involved in patient care. All patients were followed retrospectively until their last documented visit, as recorded in the Hungarian Social Security Database.

### Statistical Analysis

Continuous variables are presented as either medians with interquartile ranges (IQRs) or means with standard deviations, depending on the data distribution. Categorical variables are expressed as absolute frequencies (n) and percentages (%). The Shapiro–Wilk test was used to assess distribution normality. The Mann–Whitney *U* test was used to compare continuous variables between 2 independent groups. Categorical variables were analyzed using the χ^2^test or Fisher exact test as appropriate. To explore the associations between independent predictors and the primary outcome, a multivariable logistic regression analysis was conducted. The maximum number of predictors was given by the 1–10 rule-of-thumb. Predictors were selected based on clinical plausibility and included sex, age, presence of fever, serum CRP and procalcitonin (PCT) at diagnosis, culture positivity and *S. aureus* positivity. Continuous variables were tested for linearity-in-the-logit by the Box-Tidwell test. The model was fitted via the maximum-likelihood method. No *post hoc* variable selection or interaction terms were included. Additional statistical measurements of model calibration and discrimination can be found in Figures, Supplemental Digital Content 1, 2 and 3, https://links.lww.com/INF/G510. Odds ratios with 95% confidence intervals are reported for each predictor. A *P* value <0.05 was considered to indicate statistical significance for all tests. Calculations were performed via SPSS 23.0. The study adheres to the STROBE Statement (STrengthening the Reporting of OBservational studies in Epidemiology, www.strobe-statement.org).

## RESULTS

### Baseline Characteristics

The patients’ baseline characteristics are summarized in Table 1. A total of 97 children were enrolled, with the following distribution across centers: 55, 15, 11, 7, 5 and 4 children. There was a male predominance (67/97, 69.1%), and the median age was 6.8 years. Among the participants, 49.5% (48/97) were diagnosed with AHO, 28.9% (28/97) with SA and 21.6% (21/97) had concurrent AHO + SA. Most of the patients presented acutely (89.7%, 87/97). No statistically significant differences were observed between the subgroups in terms of age, sex, symptom duration or BJI classification. The most common clinical symptoms at diagnosis included limb/joint pain (89.7%), fever (77.3%) and joint movement restriction (74.2%). Fever was significantly more prevalent in the CP subgroup (89.8% vs. 57.9%, *P* = 0.001). Among patients with AHO, the femur (18.6%) and tibia (17.5%) were the most frequently affected sites. Notably, femoral involvement was significantly more common in the CP group than in the CN group (*P* = 0.034). In terms of laboratory results at diagnosis, the serum CRP and PCT levels were significantly higher in the CP subgroup. The overall diagnostic yield of radiologic imaging was 32.9% for plain radiography, 80.2% for ultrasonography and 97% for MRI, with no significant differences observed between the CP and CN subgroups.

**TABLE 1.** Baseline Characteristics of Included Patients

Parameter	Total (n = 97)	Culture Positivity (n = 59)	Culture Negativity (n = 38)	*P* value
Age (yr, median (IQR))	6.80 (0.79;10.69)	6.80 (0.79;9.54)	6.54 (1.46;11.64)	0.813
Age cohorts (n, %)				
<1 yr	25 (25.8)	16 (27.1)	9 (23.7)	0.706
1–5 yr	19 (19.6)	10 (16.9)	9 (23.7)	0.415
6–18 yr	53 (54.6)	33 (55.9)	20 (52.6)	0.750
Male sex (n, %)	67 (69.1)	45 (76.3)	22 (57.9)	0.056
No. of patients receiving antibiotics before admission (n, %)	21 (21.6)	9 (15.3)	12 (31.6)	0.057
Length of symptoms prior to admission (d, median (IQR))	4 (2;6)	4 (2;6)	3 (1;5.75)	0.262
Classification according to anatomy (n, %)				
AHO	48 (49.5)	30 (50.8)	18 (47.4)	0.738
SA	28 (28.9)	17 (28.8)	11 (28.9)	0.989
AHO + SA	21 (21.6)	12 (20.3)	9 (23.7)	0.696
Classification according to symptom length (n, %)				
Acute	87 (89.7)	54 (91.5)	33 (86.8)	
Subacute	10 (10.3)	5 (8.5)	5 (13.2)	0.507
Clinical symptoms at diagnosis (n, %)				
Fever	75 (77.3)	53 (89.8)	22 (57.9)	**0.001**
Limb or joint pain	87 (89.7)	55 (93.2)	32 (84.2)	0.255
Joint movement restriction	72 (74.2)	44 (74.6)	28 (73.7)	0.936
Limping	49 (50.5)	31 (52.5)	18 (47.4)	0.824
Swelling of a limb or joint	65 (67.0)	38 (64.4)	27 (71.1)	0.446
Erythema of a limb or joint	34 (35.1)	20 (33.9)	14 (36.8)	0.786
Vomiting	14 (14.4)	12 (20.3)	2 (5.3)	0.072
Difficulty feeding	16 (16.5)	10 (16.9)	6 (15.8)	0.867
Irritability	14 (14.4)	11 (18.6)	3 (7.9)	0.235
Laboratory results at diagnosis (median (IQR))				
White blood cell count (×10^9^/L)^*^	12.12 (9.0;17.40)	12.46 (8.98;19.94)	12.00 (9.00;14.00)	0.361
Absolute neutrophil cell count (×10^9^/L)^†^	7.50 (5.50;12.10)	8.40 (5.78;13.20)	6.22 (4.15;9.71)	0.079
Absolute lymphocyte count (×10^9^/L)^†^	2.17 (1.21;4,05)	1.90 (0.84;3.75)	2.82 (1.68;5.68)	0.062
Serum C-reactive protein (mg/L)^†^	85 (27;145)	100 (44;150)	43 (13;142)	**0.044**
Serum procalcitonin (ng/mL)^*^	0.87 (0.19;4.66)	2.13 (0.42;5.16)	0.29 (0.09;1.15)	**0.012**
Erythrocyte sedimentation rate (mm/h)^†^	44 (21;64)	46 (23;67)	34 (15;56)	0.294
Radiograph imaging performed^‡^ (n, %)	73 (75.3)	40 (67.8)	33 (86.6)	**0.034**
Radiograph imaging positivity^§^ (n, %)	24 (32.9)	11 (27.5)	13 (39.4)	0.240
Ultrasonography performed^‡^ (n, %)	86 (88.7)	54 (91.5)	32 (84.2)	0.267
Ultrasonography positivity^§^ (n, %)	69 (80.2)	46 (85.2)	23 (71.9)	0.212
Magnetic resonance imaging performed^‡^ (n, %)	66 (68.0)	43 (72.9)	23 (60.5)	0.270
Magnetic resonance imaging positivity^§^ (n, %)	64 (97.0)	41 (95.3)	23 (100)	0.539
Magnetic resonance imaging alterations^¶^ (n, %)				
Soft tissue abnormality (not otherwise specified)	38 (59.4)	22 (53.7)	16 (69.6)	0.508
Cellulitis	9 (14.1)	5 (12.2)	4 (17.4)	0.725
Myositis	16 (25.0)	7 (17.1)	9 (39.1)	0.097
Subperiosteal abscess	12 (18.8)	8 (19.5)	4 (17.4)	0.749
Intraosseal abscess	7 (10.9)	5 (12.2)	2 (8.7)	0.694
Intraosseal necrosis	3 (4.7)	3 (7.3)	0	0.274
Deep venous thrombosis	3 (4.7)	3 (7.3)	0	0.274
Bone sequestrum	3 (4.7)	3 (7.3)	0	0.274
Bone affected (n, %)				
Humerus	9 (9.3)	4 (6.8)	5 (13.2)	0.308
Ulna	2 (2.1)	1 (1.7)	1 (2.6)	1
Radius	1 (1.0)	0	1 (2.6)	0.392
Femur	18 (18.6)	15 (25.4)	3 (7.9)	**0.034**
Tibia	17 (17.5)	11 (18.6)	6 (15.8)	0.718
Fibula	4 (4.1)	1 (1.7)	3 (7.9)	0.296
Toe	1 (1.0)	0	1 (2.6)	0.392
Hip bone	9 (9.3)	6 (10.2)	3 (7.9)	1
Calcaneus	1 (1.0)	1 (1.7)	0	1
Clavicle	4 (4.1)	1 (1.7)	3 (7.9)	0.296
Lumbar spine	0	0	0	-
Frontal bone	1 (1.0)	1 (1.7)	0	1
Temporal bone	1 (1.0)	0	1 (2.6)	0.392
Zygomatic bone	1 (1.0)	0	1 (2.6)	0.392
Joint affected (n, %)				
Glenohumeral joint	6 (6.2)	3 (5.1)	3 (7.9)	0.676
Humeroulnar joint	1 (1.0)	1 (1.7)	0	1
Acetabulofemoral joint	11 (11.3)	8 (13.6)	3(7.9)	0.519
Tibiofemoral joint	8 (8.2)	7 (11.9)	1 (2.6)	0.143
Talocrural joint	5 (5.2)	3 (5.1)	2 (5.3)	1
Sacroileacal joint	1 (1.0)	0	1 (2.6)	0.392
Sternoclavicular joint	4 (4.1)	3 (5.1)	1 (2.6)	1

A *P* value <0.05 was considered to indicate statistical significance for all tests.

* Values are reported as median and IQR.

† Values are reported as mean ± SD.

‡ Ratios are given relative to the number of the total cohort.

§ Ratios are given relative to the number of imaging studies performed.

¶ Ratios are given relative to the number of positive imaging studies.

### Microbiologic Characteristics

The microbiologic characteristics are summarized in Table 2. An identifiable causative microorganism was detected in 60.8% of the patients (59/97). Blood cultures were obtained from 84 patients (86.6%), yielding positive results in 45 patients (53.6%). Intraoperative specimens were collected from 62 patients (63.9%), with a culture-positivity rate of 74.2% (46/62). In 23.7% of CP cases, blood cultures were negative and the intraoperative specimen represented the sole source of pathogen identification. The most common causative pathogen was *S. aureus* (71.18%, 42/59), followed by *S. pyogenes* (11.86%, 7/59), *Salmonella* species (6.78%, 4/59) and *Serratia marcescens* (3.39%, 2/59). As *S. marcescens* occurs more frequently among immunocompromised patients, immunologic testing was performed in both cases, without confirmable presence of immunodeficiency. In the case of 1 child, 2 bacteria were considered pathogens at the same time (*S. aureus*, *S. marcescens*).

**TABLE 2.** Causative and Contaminant Organisms, Isolated From Clinical Samples

Parameter	Blood Culture (n, %)	Intraoperative Bone Specimen (n, %)	Total Of Identified Organisms^*^ (n, %)
Cultures performed per total cohort	84 (86.6)	62 (63.9)	n.r.
Positive cultures per cultures performed	45 (53.6)	46 (74.2)	n.r.
Causative organisms^†^			
*Staphylococcus aureus*	30 (66.7)	33 (71.7)	42 (43.3)
*Streptococcus agalactiae*	1 (2.2)	0	1 (1.0)
*Streptococcus pneumoniae*	0	1 (2.2)	1 (1.0)
*Streptococcus pyogenes*	5 (11.1)	6 (13.0)	7 (7.2)
*Salmonella* spp.	2 (4.4)	2 (8.7)	4 (4.1)
*Serratia marcescens*	2 (4.4)	0	2 (2.1)
*Cutibacterium acnes*	0	1 (2.2)	1 (1.0)
*Chryseobacterium* sp.	1 (2.2)	0	1 (1.0)
*Klebsiella pneumoniae*	0	1 (2.2)	1 (1.0)
Contaminant organisms^†^			
*Bacillus cereus*	1 (2.2)	1 (2.2)	2 (2.1)
*Corynebacterium coyleae*	1 (2.2)	0	1 (1.0)
*Kocuria kristinae*	1 (2.2)	0	1 (1.0)
*Micrococcus luteus*	1 (2.2)	0	1 (1.0)
*Staphylococcus epidermidis*	3 (6.7)	1 (2.2)	4 (4.1)
*Staphylococcus hominis*	1 (2.2)	0	1 (1.0)
*Staphylococcus warneri*	1 (2.2)	0	1 (1.0)

n.r. indicates not relevant.

* Ratios are reported as the number of all isolates of the given bacterium per the number of patients in the cohort (n = 97). In the event of a pathogen being identified in both a blood culture and an intraoperative specimen, it was counted as one in the total cohort.

† Ratios are reported as the number of all isolates of the given bacterium per the number of all culture-positive cases of the given clinical sample.

### Clinical Outcomes and Complications

**FIGURE 1.**
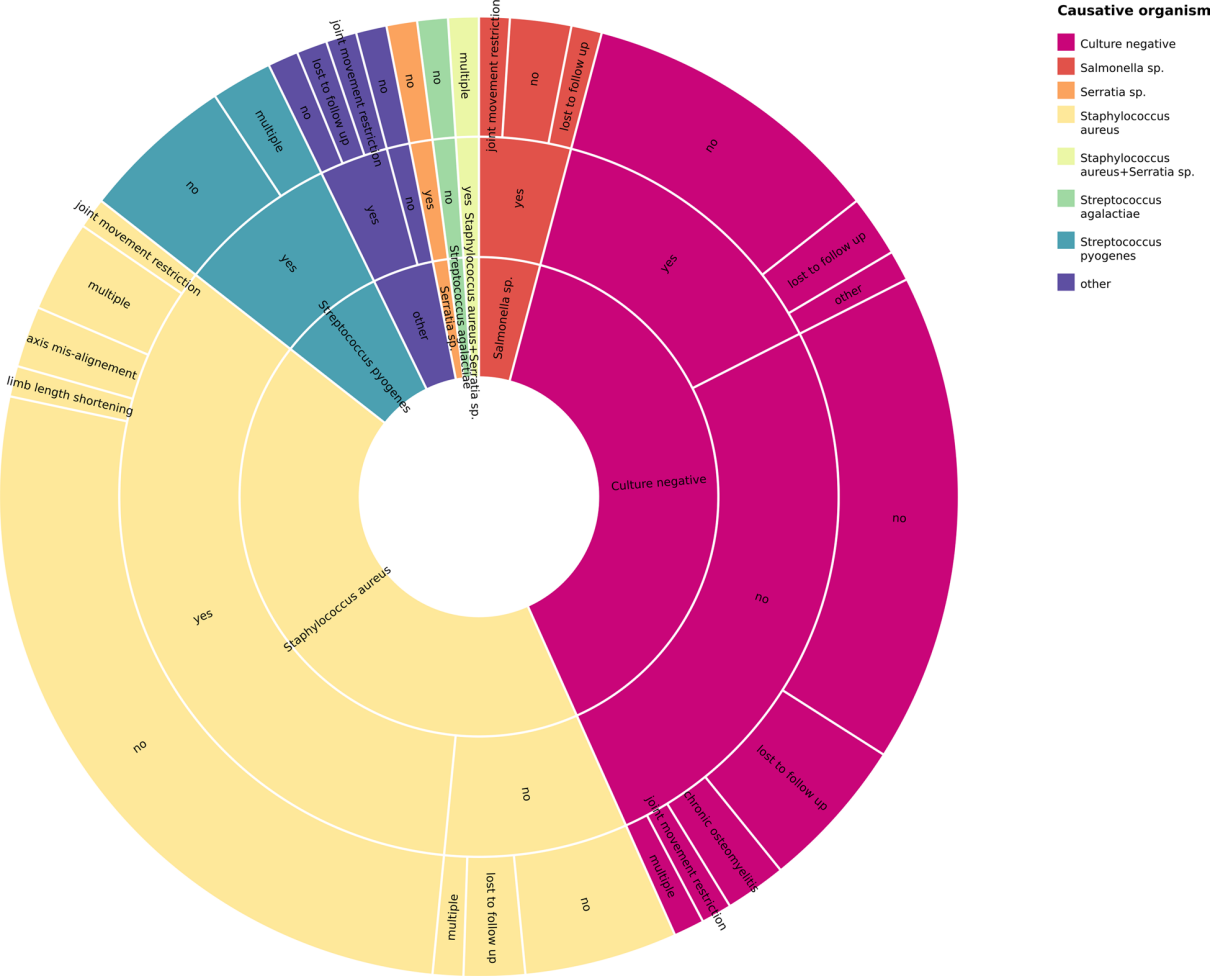
Sunburst diagram showing the connection between the causative organism (inner ring), the requirement for surgery (middle ring) and long-term complications (outer ring)

The short- and long-term outcomes are summarized in Table 3. At 30 days postdiagnosis, a significantly greater proportion of children in the CP subgroup than in the CN subgroup required surgical intervention (79.7% vs. 34.2%, *P* = 0.001). The overall incidence of short-term complications was also significantly greater in the CP group (*P* = 0.031). While the rate of infectious complications did not differ significantly, epiphyseal injury was more common in the CP subgroup (35.6% vs. 13.2%, *P* = 0.019). The requirement for additional surgical procedures to achieve source control was also greater among CP patients (18.6% vs. 2.6%, *P* = 0.025). Among patients who needed reoperation, the most identified pathogen was MSSA, which was found in 9 of 14 patients (64.3%). No statistically significant differences were observed in the incidence of long-term complications between the 2 subgroups (Fig. 1). In the CP group, long-term complications occurred in 26% of the patients (13/59), with MSSA being the predominant pathogen (9/13, 69.2%).

**TABLE 3.** Outcome Characteristics of Included Patients

Parameter	Total (n = 97)	Culture Positivity (n = 59)	Culture Negativity (n = 38)	*P* value
Requirement of orthopaedic surgery within 30 days from diagnosis (n, %)	60 (61.9)	47 (79.7)	13 (34.2)	**0.001**
Appearance of any complication within 30 days from diagnosis (n, %)	54 (55.7)	38 (64.4)	16 (42.1)	**0.031**
Infectious short-term complications (n,%)				
Pneumonia	6 (6.2)	5 (8.5)	1 (2.6)	0.660
Endocarditis	0	0	0	1
Hepatic abscess	0	0	0	1
Infective myositis or pyomyositis	27 (27.8)	18 (30.5)	9 (23.7)	0.464
Venous thrombosis^*^	5 (5.2)	4 (6.8)	1 (2.6)	0.645
Prolonged bacteriemia	3 (3.1)	3 (5.1)	0	0.277
Orthopedic short-term complications (n, %)				
Two or more bones involved	11 (11.3)	5 (8.5)	6 (15.8)	0.331
Presence of physeal injury	26 (26.8)	21 (35.6)	5 (13.2)	**0.019**
Presence of pathologic fracture	2 (2.1)	0	2 (5.3)	0.151
Appearance of any complication during follow-up from diagnosis^†^ (n, %)				
Any complications	18 (23.7)	13 (26)	5 (19.2)	0.58
Pathologic fracture	1 (1.32)	1 (2)	0	1
Limb length shortening	6 (7.89)	5 (10)	1 (3.85)	0.657
Axis misalignment	5 (6.58)	4 (8)	1 (3.85)	0.655
Joint movement restriction	9 (11.84)	8 (16)	1 (3.85)	0.153
Chronic osteomyelitis	8 (10.52)	6 (12)	2 (7.69)	0.707
Lengths of antibiotic therapy (d, median (IQR))				
Parenteral	11 (8;19)	13 (9;20)	9 (6;18)	**0.016**
Oral	20 (13;28)	22 (14;30)	19 (7;22)	0.066
Total	29 (21;42)	32 (22;44)	26 (13;31)	**0.002**
Switching from parenteral to per os antibiotics (n, %)	81 (83.5)	52 (88.1)	29 (76.5)	0.638
Length of hospital stay (d, median (IQR))	13 (8;23)	15 (10;24)	9 (6;23)	**0.032**
Patients lost to follow-up (n, %)	21 (21.6)	9 (15.3)	12 (31.6)	0.07
Median time of follow-up (months, median (IQR))	11 (8;17)	2.5 (8;20)	10 (7;15)	0.062

A *P* value <0.05 was considered to indicate statistical significance for all tests.

* All cases with venous thrombosis were associated with the underlying BJI condition.

† Only including cases with evaluable follow-up.

The outcome of the 30-day surgical intervention was modelled via multivariable logistic regression (Table 4). After adjusting for all covariates, the presence of a positive culture was significantly associated with an increased likelihood of early surgery (OR = 196.5; 95% CI 2.04–19111.1; *P* = 0.024). The overall model fit was acceptable (pseudo-*R*² = 0.44; log-likelihood ratio *P* = 0.001).

**TABLE 4.** Multivariate Logistic Regression Modeling of the Primary Outcome

	Requiring Surgery Within 30 Days(n = 62)	Not Requiring Surgery Within 30 Days (n = 35)	Adjusted Odds Ratio	95% Confidence Interval	*P* value
Male sex (n, %)	43 (69.36)	24 (68.57)	0.36	0.03–5.88	0.475
Age (months, mean ± SD)	80.79 ± 57.89	75.02 ± 71.62	1.01	0.99–1.03	0.304
Fever at diagnosis (n, %)	51 (82.26)	24 (68.57)	0.05	0,0006–3.45	0.162
Serum C-reactive protein at diagnosis (mg/L, mean ± SD)	121.2 ± 98.34	73.46 ± 66.77	1.02	1–1.04	0.093
Serum procalcitonin at diagnosis (ng/ml, mean ± SD)	6.53 ± 18.54	2.4 ± 5.85	0.99	0.94–1.07	0.99
Culture positivity (n, %)	49 (79.03)	10 (28.57)	196.5	2.04–19111.1	0.024
*S. aureus* positivity (n, %)	34 (54.84)	8 (22.86)	0.19	0.01–3.84	0.282

## DISCUSSION

### Major Findings of Our Study

This was the first multicenter study in Hungary to investigate the clinical characteristics of pediatric BJIs, with a specific focus on differences between CP and CN subgroups. Within 30 days of diagnosis, a significantly greater proportion of children in the CP group than in the CN group underwent surgical intervention and experienced short-term complications. Additionally, the CP group had a longer duration of hospitalization and antibiotic therapy. No significant differences were observed between the groups regarding long-term sequelae.

### Previous Findings From the Literature

The findings of our study are largely consistent with the literature, particularly with respect to clinical characteristics such as a median age of approximately 6 years, male predominance and frequent involvement of the long bones of the lower extremities.^12^ In the present study, the median serum CRP and PCT levels were significantly greater in the CP subgroup. These findings align with those of a retrospective study conducted by Jia et al.^4^, which included 117 pediatric patients with AHO. In their analysis, elevated CRP levels were more frequently observed in the CP group (95.8%) than in the CN group (90.5%). Moreover, children in the CN group were admitted later in the disease course and presented with lower fever rates, white blood cell counts, CRP levels and D-dimer levels, suggesting that CN osteomyelitis may represent a relatively milder clinical phenotype.

The findings of the present study are also consistent with the results of the largest pediatric cohort that has been studied to date. In their study, Williams et al.^13^ examined a total of 390 children, of whom 51.1% were classified as part of the CP group. It was observed that 70% of cases within this group were attributed to *S. aureus*. In the study, the CN group demonstrated lower concentrations of CRP, shorter durations of fever and reduced hospital length of stay, as well as both parenteral and total antibiotic days.

In pediatric BJIs, 24%–68% of AHO cases and 21%–55% of SA cases remain CN despite efforts at identification.^3^ In a study by Saavedra-Lozano et al.^14^, 55% of cultures were positive, with 37% and 75% of blood and surgical cultures becoming positive, respectively, highlighting the superior diagnostic yield of invasive sampling. Similarly, Jiun-An Chen et al.^2^ reported an overall culture-positivity rate of 52.9%, with 33.3% of blood cultures yielding a pathogen and 60.6% of infectious site cultures becoming positive. These findings are consistent with our results, although we observed higher positivity rates for both blood and intraoperative cultures. Our findings support the conclusion that invasive sampling methods offer a higher diagnostic yield than blood cultures alone.

The majority of published studies have identified *S. aureus* as the most prevalent pathogen in CP BJIs, with reported rates ranging from 67.6% to 85.5%.^2^ In our study, *S. aureus* accounted for 71.2% of the CP cases, which is consistent with previously published data. However, the prevalence of methicillin-resistant *S. aureus* (MRSA) varies considerably across countries, ranging from approximately 12% to 44.7%.^2^ According to the most recent European guidelines, empirical antibiotic regimens should include MRSA coverage if the local prevalence of community-acquired MRSA exceeds 10%–15%.^11^ Before this multicenter study, no reliable data were available on the prevalence of MRSA in pediatric BJI patients in Hungary. Our findings indicate that MRSA accounted for only 2.4% of all *S. aureus* isolates, suggesting that routine empirical MRSA coverage is likely unnecessary in our setting. In comparison, *K. kingae*, a pathogen typically associated with a milder clinical course and predominantly affecting children under 48 months of age, is increasingly recognized in many countries through molecular diagnostic techniques.^15^ Unfortunately, no data are currently available regarding the prevalence of *K. kingae* infections in Hungary.

The duration of total therapy is estimated to be approximately 2–3 weeks for SA and 3–4 weeks for AHO, according to the European guideline.^11^ The duration of antibiotic therapy in our study was approximately equivalent, with a significantly shorter duration in the CN group and with a shorter length of hospital stay. This finding aligns with the conclusions of other studies, which have indicated that patients diagnosed with CN BJIs exhibit a reduced need for intravenous antibiotic treatment and a comparatively shorter duration of hospitalization.^16^ However, as suggested in several recent studies, the early adoption of oral therapy or exclusive oral treatment was not a prevalent practice among healthcare providers in our country.^17^ This phenomenon can be attributed to the mounting body of evidence concerning these issues that has been published in recent years and has gradually been incorporated into clinical practice.

Recent studies, including our own, have demonstrated that patients diagnosed with CN BJIs exhibit a lower rate of surgical intervention than those with CP infections do.^2^ This observation may reflect a comparatively milder disease course in CN patients. In our cohort, the presence of a positive culture was associated with an increased likelihood of early surgery. According to the literature, 9%–12% of children with BJIs develop long-term orthopedic sequelae, including joint deformity, restricted range of motion, avascular necrosis, chronic osteomyelitis, appendicular growth arrest and pathologic fractures.^18^ Although several studies have reported a lower incidence of long-term complications among CN patients, our findings did not reveal a statistically significant difference between the 2 subgroups.^2^

### Limitations of Our Study

Our study has several limitations. First, as a retrospective analysis, residual bias might have confounded the results, despite efforts to ensure data consistency. Second, the identification of CN cases may, in part, reflect the absence of molecular diagnostic techniques, which could have improved pathogen detection. Finally, the incidence of certain complications may have been underestimated because of some variability in the duration of follow-up across participating centers.

## CONCLUSIONS

This first multicenter study in Hungary comparing CP and CN pediatric BJIs revealed that CN cases were associated with fewer early complications, reduced surgical intervention, and shorter antibiotic treatment and hospital stays, suggesting a milder disease course.

## Supplementary Material


