# Supplementary material for: Clinical Characteristics and Outcomes of Culture-positive Versus Culture-negative Bone and Joint Infections in Pediatric Patients: A 10-Year Multicenter Study From Hungary
Source: Pediatr Infect Dis J. 2026 Jan 5;45(7):565–71. doi: 10.1097/INF.0000000000005137 (PMC13232703; doi:10.1097/INF.0000000000005137)

SUPPLEMENTAL DIGITAL CONTENT 1. ROC curve. The model demonstrated good discrimination with an area under the ROC curve of 0.88, indicating a good discrimination of the model between patients who required surgery and those who did not. The graph was generated with ChatGPT-4.0.

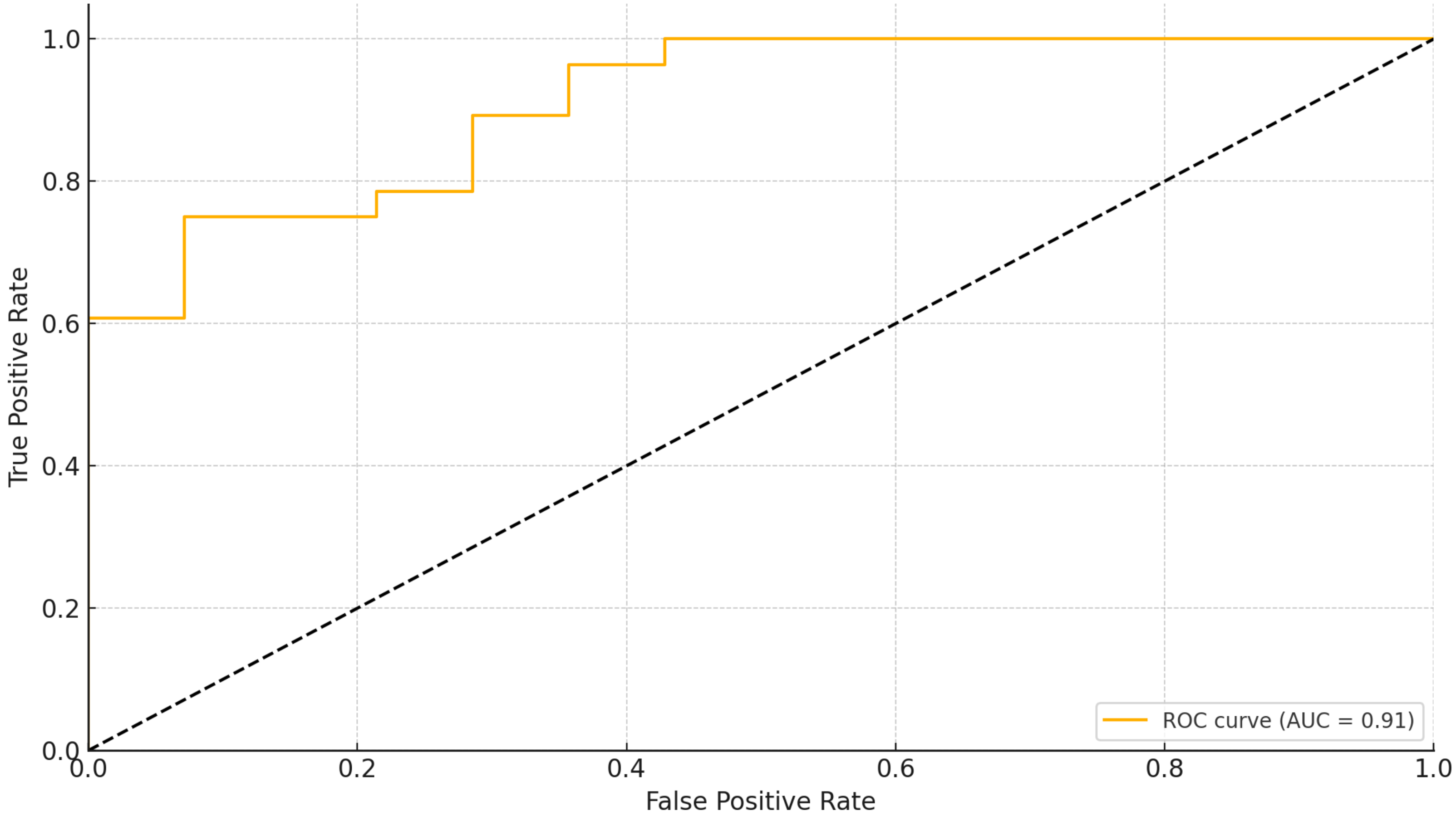

SUPPLEMENTAL DIGITAL CONTENT 2. Calibration plot. Calibration analysis showed good agreement between predicted and observed outcomes. Model performance metrics included 81% accuracy, 83% precision, and 89% recall for correctly predicting cases requiring early surgery. The graph was generated with ChatGPT-4.0.

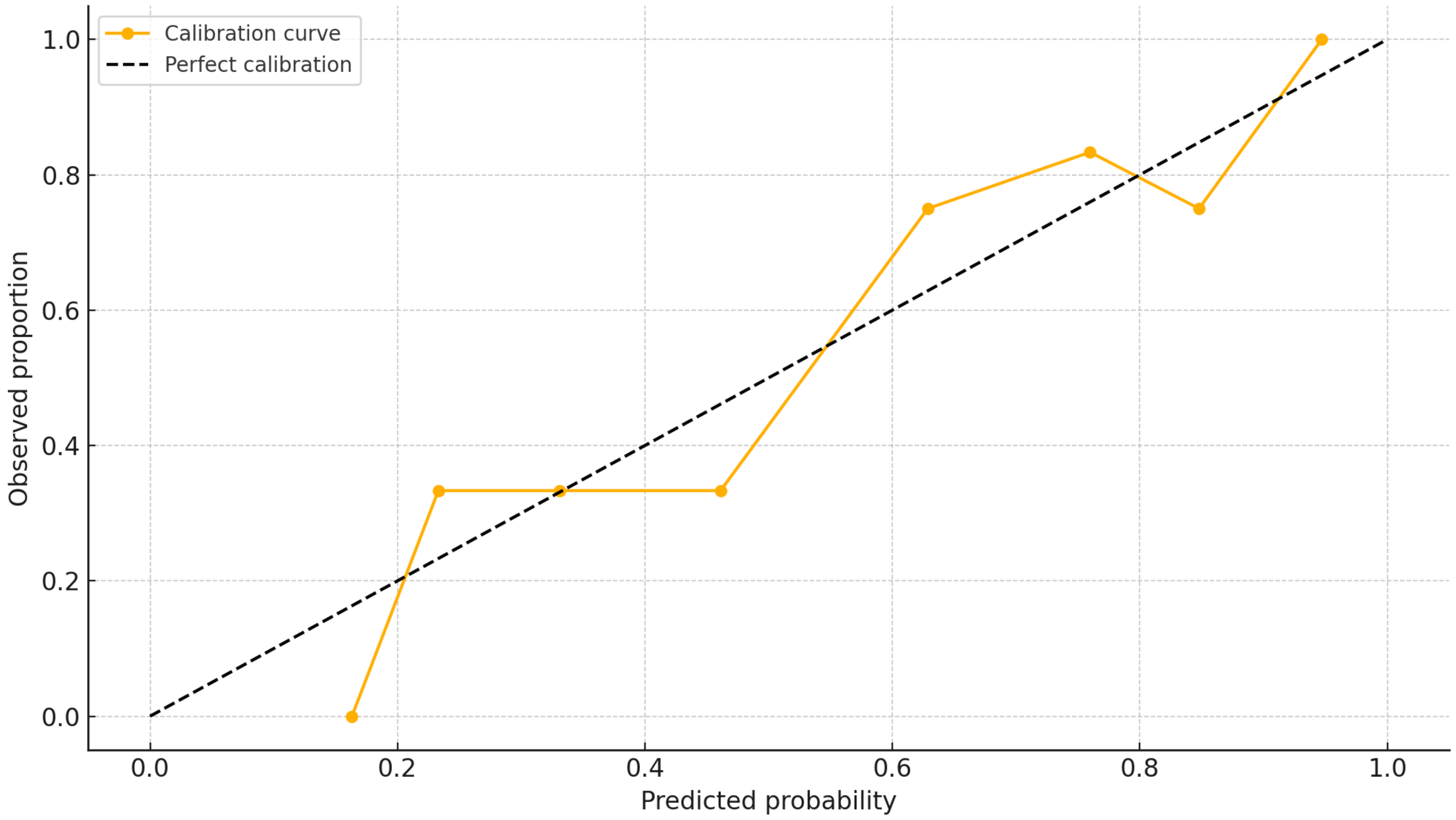

SUPPLEMENTAL DIGITAL CONTENT 3. Decision curve analysis. Decision curve analysis demonstrated a net clinical benefit of using the multivariable model across a threshold probability range of approximately 0.2 to 0.8. The graph was generated with ChatGPT-4.0.

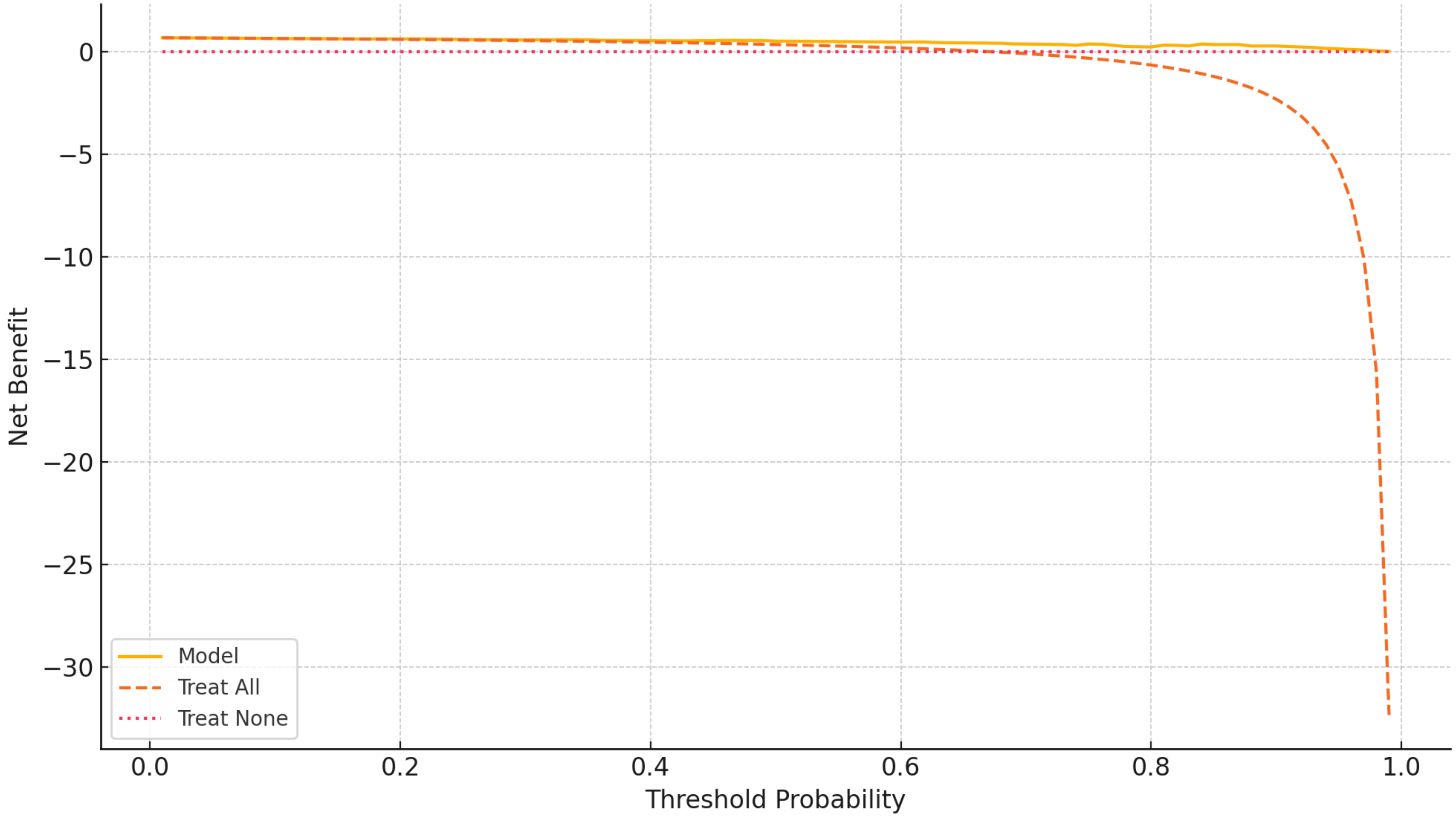

Supplement: Supplementary file 1 [file inf-45-0565-s001.pdf]
